# Signaling pathways induced by serine proteases to increase intestinal epithelial barrier function

**DOI:** 10.1371/journal.pone.0180259

**Published:** 2017-07-03

**Authors:** Kelcie A. Lahey, Natalie J. Ronaghan, Judie Shang, Sébastien P. Dion, Antoine Désilets, Richard Leduc, Wallace K. MacNaughton

**Affiliations:** 1 Department of Physiology and Pharmacology, University of Calgary, Calgary, Alberta, Canada; 2 Département de Pharmacologie, Université de Sherbrooke, Sherbrooke, Québec, Canada; University of Central Florida, UNITED STATES

## Abstract

Changes in barrier function of the gastrointestinal tract are thought to contribute to the inflammatory bowel diseases Crohn’s disease and ulcerative colitis. Previous work in our lab demonstrated that apical exposure of intestinal epithelial cell lines to serine proteases results in an increase in transepithelial electrical resistance (TER). However, the underlying mechanisms governing this response are unclear. We aimed to determine the requirement for proteolytic activity, epidermal growth factor receptor (EGFR) activation, and downstream intracellular signaling in initiating and maintaining enhanced barrier function following protease treatment using a canine intestinal epithelial cell line (SCBN). We also examined the role of phosphorylation of myosin regulatory light chain on the serine protease-induced increase in TER through. It was found that proteolytic activity of the serine proteases trypsin and matriptase is required to initiate and maintain the protease-mediated increase in TER. We also show that MMP-independent EGFR activation is essential to the sustained phase of the protease response, and that Src kinases may mediate EGFR transactivation. PI3-K and ERK1/2 signaling were important in reaching a maximal increase in TER following protease stimulation; however, their upstream activators are yet to be determined. CK2 inhibition prevented the increase in TER induced by serine proteases. The bradykinin B(2) receptor was not involved in the change in TER in response to serine proteases, and no change in phosphorylation of MLC was observed after trypsin or matriptase treatment. Taken together, our data show a requirement for ongoing proteolytic activity, EGFR transactivation, as well as downstream PI3-K, ERK1/2, and CK2 signaling in protease-mediated barrier enhancement of intestinal epithelial cells. The pathways mediating enhanced barrier function by proteases may be novel therapeutic targets for intestinal disorders characterized by disrupted epithelial barrier function.

## Introduction

The epithelial cells lining the gastrointestinal tract provide a critical barrier to prevent damaging agents from entering the underlying tissue. There are various factors that contribute to barrier function, including Paneth and goblet cell secretions, but it is the tight junction between epithelial cells that provides a final physical barrier against paracellular movement. Decreased barrier function is a characteristic of inflammatory bowel diseases (IBD) such as Crohn’s disease and ulcerative colitis. Over 1 million Americans and 2.5 million Europeans are estimated to have IBD. These diseases incur substantial costs for health care and decrease the quality of life for those affected [1,2]. IBD is a disease of developed nations, and countries that are becoming industrialized show increased incidence of IBD [3].

While the etiology of these diseases is not completely understood, IBDs are thought to arise through an inappropriate immune response to commensal bacteria [4]. Current therapies for IBD aim to limit the immune response, and while some are effective in a subset of patients, there are issues of resistance, serious side effects and relapse of disease in others. Understanding how barrier function is modified in intestinal epithelial cells may provide new avenues for treatment of diseases characterized by changes in barrier function.

The tight junction is composed of a variety of transmembrane proteins such as occludin and members of the claudin family, and is connected to the cortical actin cytoskeleton via intracellular zona occludens (ZO) family proteins. A variety of signaling pathways can modify the structure of the tight junction and the cytoskeleton to alter barrier function. One avenue of regulation is through phosphorylation of myosin II regulatory light chain (MLC) by Rho-associated protein kinase (ROCK) or myosin light chain kinase (MLCK). These events induce reorganization and contraction of the actin cytoskeleton [5]. The force of cytoskeletal contraction is transferred to the tight junction through the intermediary ZO-1, inducing a pulling of the junction apart to increase permeability [6].

In addition to barrier regulation mediated by changes to cytoskeletal components, the tight junction can also be modulated through removal or insertion of tight junctional proteins at the membrane by vesicle trafficking [7,8]. Phosphorylation events by a variety of upstream kinases and phosphatases control the orientation and organization of tight junctional proteins in the membrane [9]. Inflammatory cytokines, such as those produced during IBD, induce an increase in permeability through modification of claudins at the junction (an increase in pore-forming claudin-2 and a decrease in “tight” claudins such as claudin-1 or claudin-4) and through increased expression and activity of MLCK [10].

While several signaling proteins have been identified as modulators of barrier function through modification of phosphorylation, this level of regulation is incompletely understood as the reported effects are cell-line and tissue-dependent [11]. In general, serine and threonine phosphorylation of tight junctional proteins such as occludin is increased as the tight junction forms, and is lost during barrier disruption, while tyrosine phosphorylation is sometimes associated with tight junction disassembly [11,12].

Signaling molecules shown to regulate tight junctions include kinases such as Src kinase, protein kinase C (PKC) isoforms, and extracellular regulated kinase (ERK), as well as phosphatases [11]. Dextran sodium sulfate (DSS) treatment of Caco-2 cells triggers JNK-mediated c-Src activation that increases tyrosine phosphorylation of occludin and ZO-1, and results in the disruption of tight junctions [13]. These events also occur in a cell model of oxidative stress, and can be blocked by the Src inhibitor, PP2. Likewise, expression of a kinase-inactive c-Src delays tight junctional disruption [14]. ERK can also regulate tight junctional components by phosphorylation events. Treatment with epidermal growth factor (EGF) activates ERK signaling that prevents disruption of occludin and ZO-1 interactions following acetaldehyde injury of Caco-2 monolayers [15].

Another kinase known to phosphorylate occludin is casein kinase 2 (CK2) [16]. This serine/threonine kinase is associated with the tight junction and directly interacts with occludin [17]. CK2 can phosphorylate specific residues (T400, T404, and S408) on the C-terminal tail of occludin. Raleigh *et al*. [18] found that inhibition of CK2 increases epithelial barrier function. Furthermore, the mutation S408A mimics the dephosphorylated occludin generated by CK2 inhibition, and caused an increased association of occludin with ZO-1, claudin-1, and claudin-2.

One well-studied group of kinases known to mediate barrier function is the PKC family. This large family of kinases is divided into three groups: the classical, novel, and atypical PKCs (cPKCs, nPKCs, aPKCs, respectively). At the plasma membrane, the nPKC isoforms δ and θ, and the aPKCs ζ and λ are closely associated with TJ proteins [11]. PKCζ binds to and phosphorylates occludin on threonine residues. Inhibition of this kinase causes redistribution of occludin and ZO-1 from the tight junction, and a decrease in barrier function [19].

The serine proteases trypsin and elastase have been shown to decrease permeability in human airway epithelial cells through a calcium-dependent mechanism, suggesting the possibility of a similar role in intestinal epithelial cells [20]. Previous work in our lab has shown that serine proteases such as trypsin, chymotrypsin, and neutrophil elastase significantly increase TER in intestinal epithelial cell lines [21]. We have also shown that the serine protease, matriptase, also increases TER [22]. Matriptase, a type II transmembrane serine protease expressed in virtually all epithelia in the body, has been shown to play important roles in both the formation and maintenance of barrier function in the gastrointestinal tract [23,24]. Together, these findings show a physiologically relevant account of serine protease-dependent maintenance and functioning of intestinal epithelial cells.

While it is known that serine proteases modulate barrier function, the mechanisms behind these changes are incompletely understood. Previous work has indicated that the increase in TER in intestinal epithelial cell lines is independent of ion secretion and protease-activated receptor 2 (PAR2), but dependent on a functional tight junction, specifically occludin [22]. However, the cell surface target(s) of these proteases regulating tight junction formation are unknown. Furthermore, while we have shown that PKCζ plays a key role in the response [21], the roles of other signaling pathways and the actin cytoskeletal modulation are unknown. We investigated several signaling pathways for their potential role in the serine protease-induced increase in TER.

## Methods

### Reagents

Porcine pancreatic trypsin (trypsin-1) was purchased from Sigma-Aldrich Canada (Oakville, Ontario; catalogue #T0303) and diluted in sterile Dulbecco’s Phosphate Buffered Saline (DPBS) (Thermo Scientific, Logan, Utah, USA). The catalytically active subunit of matriptase was purified as previously described [25]. The activity of matriptase was previously determined [22] and units of enzymatic activity compared directly to trypsin BAU/mL.

Forskolin was purchased from Alfa Aesar (Ward Hill, Massachusetts, USA) and diluted in anhydrous ethanol. Soybean trypsin inhibitor (SBTI) was purchased from Sigma-Aldrich Canada and diluted in sterile DPBS. Aprotinin was purchased from Boehringer-Mannheim (Indianapolis, Indiana, USA) and diluted in sterile water. Epidermal growth factor (EGF) was purchased from R&D Systems (Minneapolis, Minnesota, USA) and reconstituted in sterile DPBS. Bradykinin acetate salt was purchased from Sigma-Aldrich Canada and diluted in 5% acetic acid. The following inhibitors were used: PD153035 hydrochloride (Tocris Bioscience, Minneapolis, Minnesota, USA, 1037), U0126 (Promega, Madison, Wisconsin, USA, V1121), PP1 (Cayman Chemical, Ann Arbor, Michigan, USA, 14244), LY294002 (Cell Signaling Technology, Danvers, Massachusetts, USA, 9901), wortmannin (Provided by Dr. Robert Newton, University of Calgary) (Sigma-Aldrich Canada, W1628), bradykinin B(2) receptor antagonist (Sigma-Aldrich Canada, B1650), GM6001 (Tocris Bioscience, 2983), marimastat (Tocris Bioscience, 2631), and TAPI-1 acetate salt (Sigma-Aldrich Canada, SML-0739). The CK2 inhibitor, tetrabromocinnamic acid (TBCA), was purchased from Cayman Chemicals and dissolved in DMSO. Canine IFN**γ** and TNFα were purchased from R&D Systems and dissolved in sterile PBS.

Antibody sources used throughout the study are as follows: phospho-PKCζ (threonine 560) (Epitomics, Burlingame, California, USA, 2200-S), β-actin (Sigma-Aldritch A5441), and pan-myosin light chain (Rockland 600-401-938, provided by Dr. Justin MacDonald, University of Calgary). The following antibodies were from Cell Signaling Technology: phospho-Akt (serine 473) (9271), Akt (9272), phospho-p44/42 MAPK (4370), p44/42 MAPK (9102), phospho-Src family (tyrosine 416) (2101), Src (36D10) (2109), and phospho-myosin light chain (Thr18/Ser19) (3674).

### Cell culture

The canine epithelial cell line, SCBN, was provided by Dr. André Buret (University of Calgary). SCBNs were grown in Dulbecco’s Modified Eagle Medium (DMEM, high glucose) supplemented with 5% fetal bovine serum (Life Technologies, Burbury, Ontario) and 2% L-glutamine (Thermo Scientific), 1% penicillin-streptomycin (Thermo Scientific), and 5 μg/ml Plasmocin (InvivoGen, San Diego, California, USA). Cells were maintained in a 37°C humidified incubator with 5% CO_2_. Cells were seeded at 1 x 10^5^ cells/well on 1.1 cm^2^, 0.4 μM pore-size polycarbonate Snapwell^™^ or transwell semi-permeable supports and grown for 5 days. Media was replaced on the basolateral side of each well every two days. Confluence was determined by ability of the cell monolayer to hold back fluid.

### Ussing chambers

Confluent cells grown on Snapwells were mounted in Ussing chambers (Physiologic Instruments, San Diego, California, USA) to measure short circuit current (I_SC_) and transepithelial electrical resistance (TER). Cells were bathed in Krebs buffer solution (pH 7.4) consisting of 115 mM NaCl, 2 mM KH_2_PO_4_, 2.4 mM MgCl_2_*6H_2_O, 25 mM NaHCO_3_, 8 mM KCl, 1.3 mM CaCl_2_, and supplemented 10 mM glucose and 10 mM mannitol on the basolateral and apical sides, respectively. Experiments with TBCA were performed in Krebs buffer without MgCl_2_. Tissue was voltage clamped to 0 V and unclamped every 20 seconds and a 5 mV potential difference applied using a voltage-clamp apparatus (VCC MC8, Physiologic Instruments). Change in current was measured using a digital data acquisition system (BioPac, Goleta, CA) and TER calculated using Acquire and Analyze software (Physiologic Instruments). Cell viability was determined at the end of each Ussing chamber experiment through measurement of the change in short circuit current (I_sc_) induced by 10 μM forskolin (Alfa Aesar, Ward Hill, MA).

### Inhibition of catalytic activity in Ussing chambers

The requirement of proteolysis by trypsin to initiate the protease-mediated increased TER was investigated using a catalytic inhibitor of the protease. The concentrations of SBTI and aprotinin needed to inhibit trypsin and matriptase, respectively, were determined using a protease activity assay with the Boc-QAR-AMC fluorogenic substrate (Bachem, Torrance, California, USA). SCBN cells mounted in Ussing chambers were incubated apically with SBTI for 30 minutes then stimulated with trypsin to confirm a requirement for proteolysis by trypsin in initiating protease-induced increased TER. The involvement of catalytic activity by trypsin or matriptase to sustain the protease-induced increased TER was investigated via the addition of SBTI post-trypsin stimulation or aprotinin post-matriptase stimulation. SCBNs mounted in Ussing chambers were treated apically with SBTI or aprotinin 10 minutes (at the beginning of the sustained phase) after trypsin or matriptase stimulation. The ability of SBTI to inhibit the response to serine proteases was also determined 30 minutes (when the sustained phase had been established) after trypsin stimulation.

### Analysis of chamber data

Percent peak TER was determined as an indicator of the ability of the cells to increase TER in the initiation phase, and percent change in TER was used to determine the effect of an inhibitor on the sustained phase, usually 15–30 minutes after the addition of trypsin. Effects of inhibitors on the protease-induced increase in TER were determined by measuring the change in TER from baseline with inhibitor, and taking it as a percentage of change in TER in cells treated with vehicle.

### Epidermal growth factor and TER

SCBNs mounted in Ussing chambers were stimulated apically with increasing doses of EGF to confirm the growth factors’ ability to increase TER in intestinal epithelial cells. To ensure SBTI was selectively inhibiting the catalytic activity of trypsin, the effect of SBTI pre-treatment on an unrelated molecule that increases TER was investigated.

### RT-PCR for ErbB family members

SCBNs grown on transwell inserts were lysed using the Qiagen Quick-Start Protocol (Qiagen Inc., Mississauga, Ontario). mRNA was isolated using the Qiagen^®^ RNeasy Mini Kit Protocol (Qiagen Inc.) as per manufacturer’s instructions. cDNA was made using Invitrogen Superscript^™^ III Reverse Transcriptase (Carlsbad, California, USA) according to manufacturer’s protocols. Primers for the ErbB receptor family (EGFR, ErbB2, ErbB3, and ErbB4) were designed using Primer-BLAST. Primers were designed to span exon-exon junctions and are as follows: EGFR forward: GGT GGT CCT GGG GAA TTT GG, EGFR reverse: CAG CGC CTT GTA GTA TTT CAT GC (product 266 bp); ErbB2 forward: GCA CCC AAG TGT GCA CCG, ErbB2 reverse: TAG CCT CTG CAG TGG GAT CT (product 233 bp); ErbB3 forward: TAA AGT CCT TGG CTC AGG CG, ErbB3 reverse: GGC CAG CAT ATG ATC CGT CA (product 151 bp); ErbB4 forward: ATG GAC CGG GAC CTG ACA A, ErbB4 reverse: TGA CTA GTG GGA CCG CTA CA (product 184 bp); Bactin forward: CGT GGG CCG CCC TAG GCA CCA, Bactin reverse: TTG GCC TTA GGG TTC AGG GGG (product 260 bp).

RT-PCR was carried out using Qiagen^®^ HotStarTaq^™^ Master Mix, according to the manufacturer’s protocols. RT-PCR was performed using the Bio-Rad T100^™^ Thermal Cycler with the following conditions: 95°C for 15 minutes, followed by 35 cycles of 95°C for 1 minute, 57°C for 1 minute, and 72°C for 1 minute. A final elongation step is performed at 72°C for 10 minutes. PCR samples were combined with 5x loading dye (xylene cyanol, 30% glycerol) and run on a 1.5% agarose gel containing 0.004% ethidium bromide. Sizes of bands were determined by comparing to the 1kb Plus DNA Ladder (Invitrogen). The gel was imaged using the Bio-Rad Chemidoc, and analysed using QuantityOne software.

### Inhibitors in Ussing chambers

Selected cell signaling proteins were investigated for their role in the initiation phase of the serine-protease induced increase in TER. Inhibitors were added apically to the cells for 15–30 minutes prior to the apical challenge of 45 BAU/mL trypsin. The EGFR inhibitor PD153035 or the CK2 inhibitor TBCA were added 20 or 15 minutes (respectively) prior to trypsin challenge, while the MMP inhibitors GM6001 and marimastat, the TACE inhibitor TAPI-1, the Src inhibitor PP1, PI3-K inhibitors LY294002 and wortmannin, the MEK1/2 inhibitor U0126, the bradykin B(2) antagonist, or the PKCζ pseudosubstrate inhibitor were added 30 minutes prior to the addition of trypsin. The concentration of PD153035 was determined based on the ability of the inhibitor to block the EGF-induced increase in TER. The concentration of bradykinin B(D) antagonist was chosen based on the ability of the inhibitor to block the basolateral bradykinin-induced increase in chloride secretion.

To determine the role of these signaling proteins in the sustained phase of the serine protease mediated increase in TER, selected inhibitors were also added apically to the cells 10 minutes post apical trypsin treatment.

### Western blotting

Confluent SCBN monolayers were washed with DPBS then serum starved for 60 minutes using SCBN media without FBS. When used, inhibitors were added during the serum starvation period. Monolayers were then stimulated apically with 45 BAU/ml trypsin, 100 ng/ml EGF, or Krebs buffer solution for control wells for 5 minutes. DMSO was used as a vehicle control for inhibitor experiments. Monolayers were washed with ice cold DPBS and 300 μl of lysis buffer were added to each well. Lysis buffer contained 100 mM NaCl, 20 mM Tris-HCl (pH 8), 0.5% Triton-X 100, 0.1% sodium dodecyl sulfate (SDS), 1 mM EDTA, 2 mM Na_3_VO_4_, and 50 mM NaF. Protease inhibitor cocktail was also added, producing a final concentration of 40 μM AEBSF, 280 nM E-64, 2.6 μM bestatin, 20 nM leupeptin, 6 nM aprotinin, and an additional 20 μM EDTA. Inserts were scraped, and cells were vortexed and frozen at -80°C overnight. Lysates were then centrifuged at 20,000 x *g* for 10 minutes at 4°C to pellet cell membrane debris, and the supernatant was collected. Whole protein quantification was done using the colorimetric DC^™^ Protein Assay Kit (Bio-Rad, Hercules, California, USA).

Protein lysates were combined with DPBS and 5X loading buffer (50 mM Tris-HCl pH 6.8, 1% SDS, 0.001% bromophenol blue, 3% glycerol and 1% β-mercaptoethanol) and were boiled for 5 minutes. Precision Plus Protein Kaleidoscope^™^ (Bio-Rad) and 15 μg of each protein sample were loaded into a Criterion^™^ 4–12% gradient, Bis-Tris polyacrylamide pre-cast gels (Bio-Rad). Following separation, proteins were transferred to 0.22 μM nitrocellulose membrane (GE Water and Process Technologies, Trevose, Pennsylvania, USA).

Membranes were washed three times for 5 minutes each in 0.1% Tween and Tris-buffered saline containing 50 mM Tris-HCl, 137 mM NaCl, and 2.7 mM KCl (TTBS). Blots were blocked in 5% non-fat dry milk or bovine serum albumin (BSA, Fisher Scientific, Ottawa, Ontario) in TTBS for 60 minutes at room temperature. Blots were incubated with primary antibody at 4°C with shaking overnight. Blots were washed and appropriate secondary antibody (goat anti-mouse or goat anti-rabbit, Jackson ImmunoResearch Laboratories, West Grove, PA) in 5% milk at 1:5000 dilution was added for 60 minutes shaking at room temperature. Blots were washed and incubated in Clarity^™^ Western Enhanced Chemiluminescent Substrate (Bio-Rad). Blots were visualized using the Kodak Image Station 4000 mm Pro and Kodak MI SE Software (Kodak, Toronto, Ontario) or Bio-Rad ChemiDoc. Densitometry for visualized bands was performed using QuantityOne^®^ 1D analysis software (Bio-Rad).

### Phos-tag western blotting

To determine the phosphorylation of myosin regulatory light chain (MLC), Phos-tag gel SDS-PAGE was utilized. This method uses a polyacrylamide-bound Mn^2+^-phosphate binding tag, which separates proteins based on their phosphorylation state. The method has previously been optimized for MLC [26]. SCBN cells were incubated in Krebs buffer to mimic Ussing chambers conditions and treated apically with trypsin or 0.5 BAU/mL matriptase for 15 minutes. Cells were collected in a lysis buffer containing 100 mM NaCl, 20 mM Tris-HCl, 1% Triton X-100, 0.5% SDS, 1 μM okadaic acid, 1 mM PMSF, 1 mM Na_3_VO_4_, and 12.5 mM NaF. Positive controls include SCBN cells treated for 30 minutes apically with 3 μM of the phosphatase inhibitor Calyculin A (Santa Cruz Biotechnology sc-2400). Protein concentration was determined and samples made as previously described.

Gels were made in Bio-Rad Criterion Empty Cassettes (1 mm thick). The stacking gel was composed of 4.5% (wt/vol) acrylamide, 0.12% *N*,*N’*,methylenebisacrylamide (30:1 acrylamide-bisacrylamide), 0.1% SDS, 125 mM Tris HCl pH 6.8, 0.06% ammonium persulfate, and 0.001% TEMED (N,N,N’,N’ tetramethylaethylenediamine). The resolving gel was composed of 11.8% (wt/vol) acrylamide, 0.34% (wt/vol) *N*,*N’*,methylenebisacrylamide (30:1 acrylamide-bisacrylamide), 40 μM Phos-tag acrylamide (Wako Chemicals, Richmond VA), 80 μM MnCl_2_, 0.1% SDS, 375 mM Tris HCl pH 8.8, 0.07% ammonium persulfate, and 0.0007% TEMED. Samples were made to 1 μg/μL and 35 μg/lane were run on 12-well gels or 15 μg/lane on 26-well gels.

Gels were run in buffer containing 0.1% SDS, 25 mM Tris, and 192 mM glycine at 20 mA/gel with the BLUeye Prestained Protein Ladder (FroggaBio, Toronto ON). Gels were removed and incubated in transfer buffer with 4 mM EDTA for 20 minutes to chelate the Phos-tag. Gels were transferred to PVDF membrane overnight at 28 mV at 4°C. The membrane was fixed in 0.5% gluteraldehyde in PBS for 20 minutes at room temperature. Membranes were washed and blocked for 1 hour in 5% milk, and incubated in primary antibody for two hours at room temperature. Blots were washed and incubated in secondary antibody for 1 hour at room temperature, and bands visualized as described previously.

### Statistics

Data analysis was performed using GraphPad Prism 6 software (La Jolla, California, USA). All analyses represent at least three independent experiments and are expressed as mean ± standard error of the mean (SEM). Data from Ussing chamber experiments were analyzed using one-way ANOVA without repeated measures when comparing more than two groups; Dunnett post-tests were used when comparing all treatments to control value only, and Tukey’s post-tests were used when comparing treatments to control and other treatment values. One-sample t-tests were used when comparing a treatment value to a hypothetical control value (100%), student t-tests were used to compare two groups. For the western blotting timecourse experiment, ratios of phosphorylated protein to total protein densitometry values were calculated and analyzed using one-way ANOVA and Dunnett post-test. For western blotting inhibitor studies, ratios of phosphorylated protein to total protein densitometry values were calculated then normalized to total fluorescence of blot to account for variation between experiments. A p value of less than 0.05 (p<0.05) was considered to be a significant difference.

## Results

### The serine protease-mediated increase in TER requires proteolytic activity

**Fig 1:**
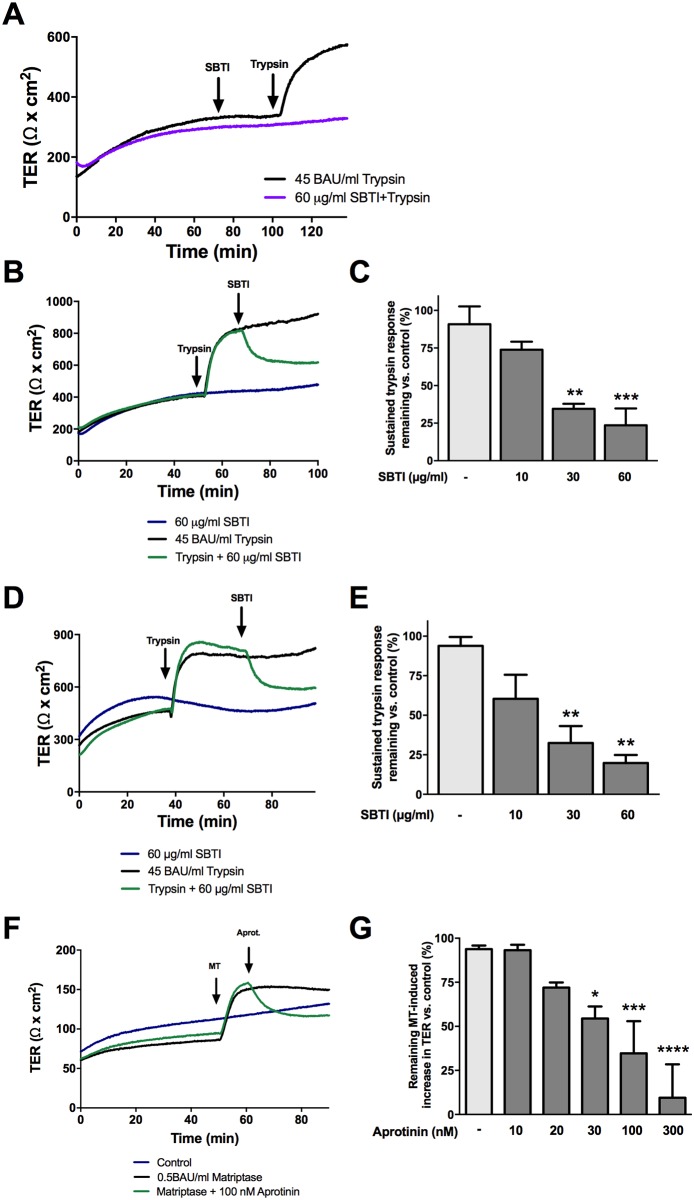
The serine protease induced increase in TER is dependent on proteolytic activity. Confluent SCBN cells were mounted in Ussing chambers and treated apically with the serine protease inhibitors SBTI or aprotinin before or after apical treatment of 45 BAU/mL trypsin or 0.5 BAU/mL matriptase, respectively. **A**. Representative tracing of cells treated with 60 μg/mL SBTI 30 minutes prior to the addition of trypsin. **B**. Representative tracing of cells treated with trypsin followed by apical treatment with 60 μg/mL SBTI after 10 minutes. **C**. The percent sustained trypsin response was determined for cells treated with SBTI 10 minutes after trypsin treatment, n = 3–4. **D**. Cells were also treated 30 minutes post trypsin addition with SBTI, and a representative tracing of 60 μg/mL SBTI is shown. **E**. Percent sustained trypsin response was determined for cells treated 30 minutes post trypsin addition, n = 3–5. **F**. Representative tracing of cells treated apically with matriptase followed by 100 nM aprotinin after 10 min. **G**. Percent sustained matriptase (MT) response was determined for cells treated 10 minutes post matriptase addition was determined, n = 3. ** p<0.01, *** p<0.001 ****p<0.0001 compared to trypsin or matriptase treated only controls by ANOVA with Dunnett’s post-hoc test.

In order to examine whether proteolytic activity was required to induce the serine protease-induced increase in TER, serine proteases were inhibited by SBTI or aprotinin during either the initiation or sustained phase of the response. Pretreatment of cells in Ussing chambers with 60 μg/mL SBTI prevented the increase in TER induced by trypsin, as demonstrated previously [21] (Fig 1A). Addition of 30 or 60 μg/mL SBTI 10 minutes post-trypsin treatment at the beginning of the sustained phase resulted in a sharp decrease in sustained TER almost immediately after its addition (Fig 1B and 1C). SBTI alone did not cause a decrease in TER in cells unchallenged by trypsin. Similar results were observed when SBTI was added 30 minutes post trypsin addition (Fig 1D and 1E), and when matriptase was inhibited by 30, 100, and 300 nM aprotinin (Fig 1F and 1G).

### Trypsin-mediated increased TER requires EGFR activation

**Fig 2:**
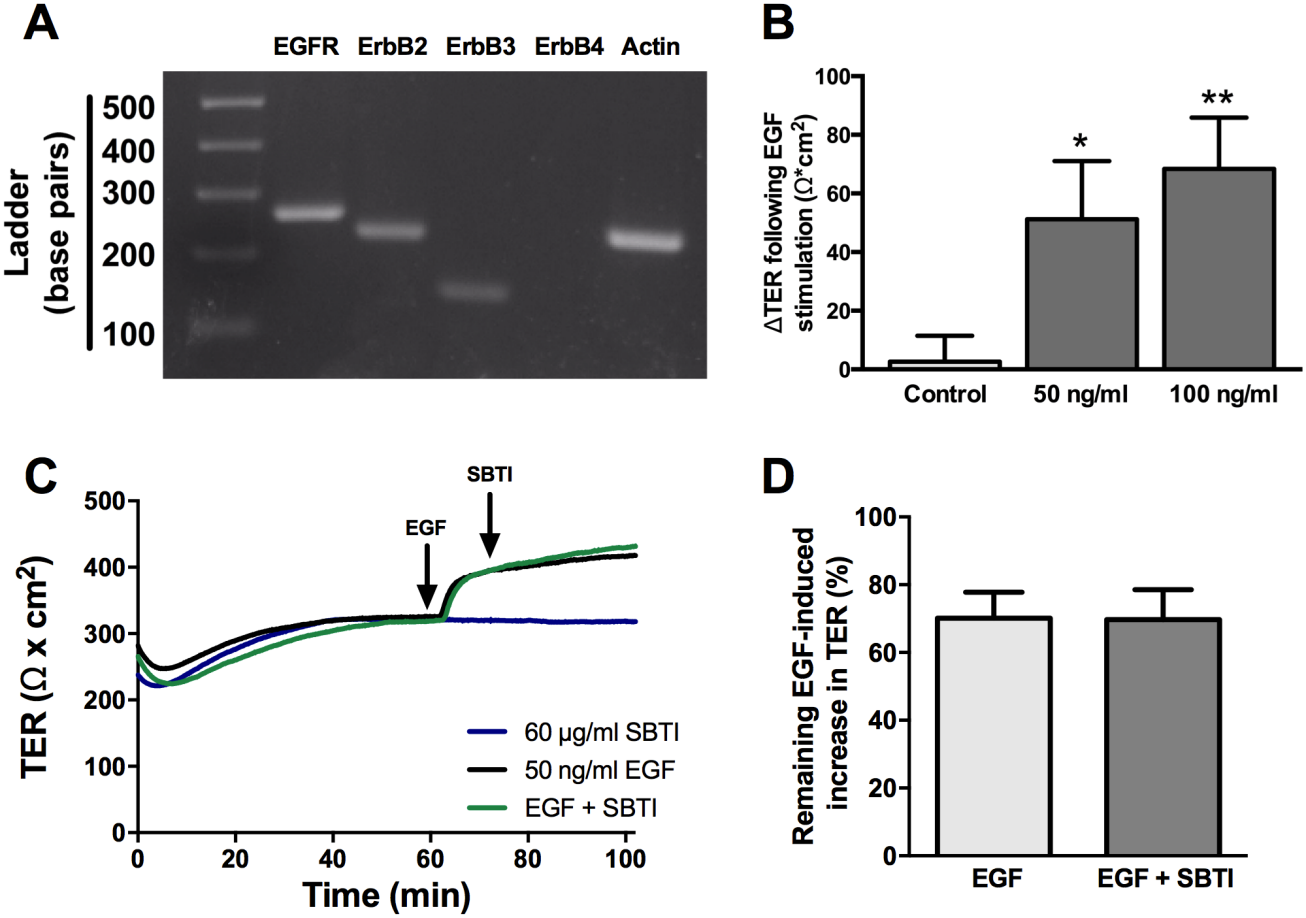
EGF induces an increase in TER in SCBN cells similar to serine proteases. **A**. SCBN cells were examined for mRNA expression of EGFR, ErbB2, ErbB3, and ErbB4 using RT-PCR. **B**. SCBN cells were mounted in Ussing chambers and treated with 50 or 100 ng/mL EGF and change in TER determined, n = 3 *p<0.05 ** p<0.01 compared to control by ANOVA with Dunnett’s post-hoc test. **C**. In Ussing chambers, SBTI was added apically to the cells 10 minutes post EGF treatment and representative tracing is shown. **D**. No significant differences were found when SBTI was added to cells treated with EGF, n = 3.

Similar to trypsin, apical addition of EGF induces an increase in TER in SCBN cells, although the change in TER is less robust. We initially validated the expression of EGFR, ErbB2, and ErbB3 mRNA in SCBN cells by RT-PCR (Fig 2A). ErbB4 was not detected. The change in TER elicited by 50 ng/mL EGF was not statistically different from that elicited by 100 ng/mL (Fig 2B), and thus 50 ng/mL EGF was used in subsequent experiments. SBTI was unable to induce a decrease in the EGF-induced increase in TER (Fig 2C and 2D).

**Fig 3:**
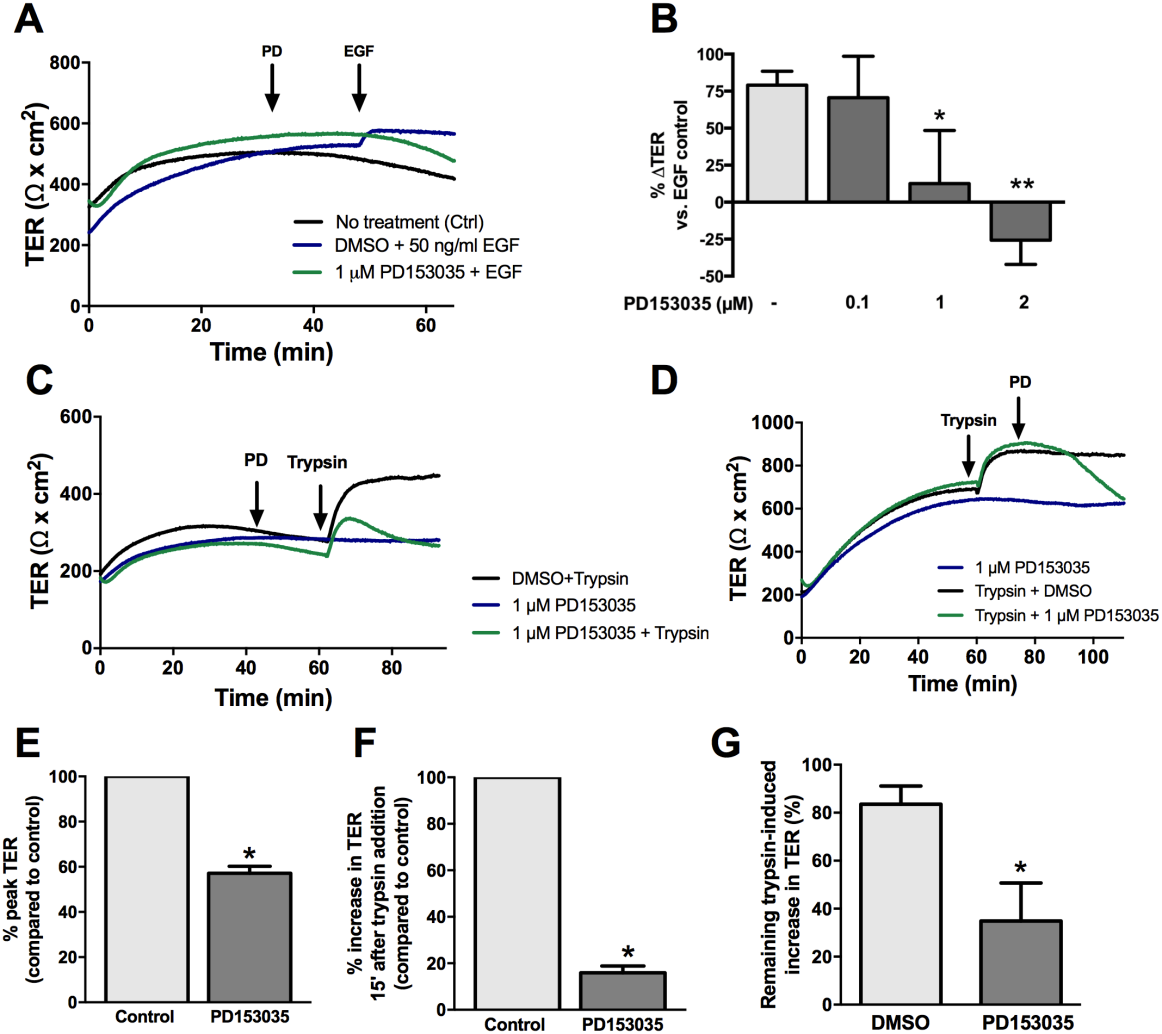
The serine protease induced increase in TER is partially dependent on EGFR. Confluent SCBN monolayers mounted in Ussing chambers were pre-treated apically with 0.1, 1, or 2 μM PD153035 or DMSO vehicle control for 20 minutes then stimulated apically with 50 ng/mL EGF. **A**. A representative tracing of n = 3. **B**. Percent change in TER was determined 15 minutes after addition of EGF. * p<0.05, ** p<0.01 compared to DMSO + EGF-treated control by ANOVA with Tukey’s post-hoc test. A concentration of 1 μM PD153035 was then used for subsequent experiments. **C**. SCBN cells were pretreated with 1 μM PD153035 for 20 minutes prior to the addition of 45 BAU/mL trypsin. A representative tracing is shown of n = 4. Percent peak TER **(E)** and the percent increase in TER 15 minutes post trypsin addition **(F)** were determined and PD153035 significantly reduced both parameters * p<0.05 as assessed by one sample T-test. **D**. PD153035 was added apically to cells after a new plateau had been reached, and a representative tracing shown of n = 6–7. **G**. The percent remaining trypsin-induced increase in TER was then determined. * p<0.05 as assessed by one sample T-test.

To determine whether trypsin induces a change in TER through EGFR activation, the EGFR tyrosine kinase inhibitor PD153035 was used. First, the lowest concentration of PD153035 that could block the EGF-induced increase in TER was determined (Fig 3A). The apical addition of 1 μM PD153035 significantly decreased the EGF-induced increase in TER without affecting baseline TER, and therefore was used in subsequent experiments (Fig 3B). Cells were either incubated with PD153035 30 minutes prior to apical trypsin treatment (Fig 3C), or PD153035 was added 10 minutes post trypsin treatment (Fig 3D). Pretreatment with PD153035 did not affect the slope of the initiation phase, but the peak increase in TER and change in TER 15 minutes post treatment were both significantly decreased (Fig 3E and 3F). EGFR inhibition during the sustained response, 10 minutes post trypsin treatment, significantly reduced the trypsin-induced increase in TER (Fig 3G).

### EGFR activation in response to trypsin occurs independently of endogenous MMP or ADAM17 cleavage

We hypothesized that serine proteases may be cleaving growth factors from the cell surface that could then act on the cell to induce an increase in TER. Activation of EGFR can occur through extracellular cleavage of a membrane-bound precursor ligand to release a soluble ligand that can then bind to and activate its cognate receptor [27]. MMPs have been implicated in this cleavage mechanism, and therefore the role of MMP-mediated EGFR transactivation in the effect of trypsin on TER was examined.

**Fig 4:**
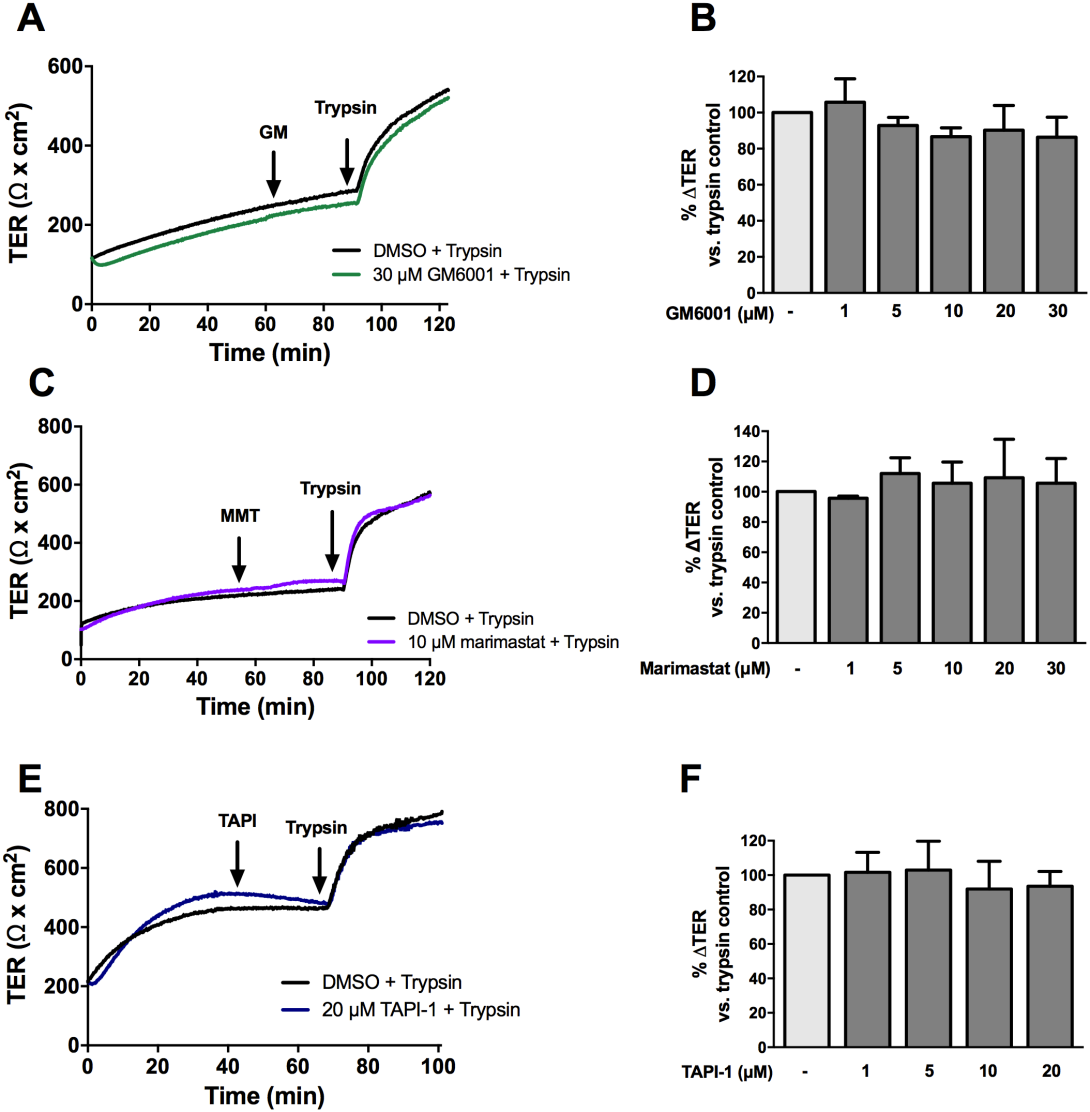
The serine protease mediated increase in TER is not dependent on MMPs. SCBN cells were mounted in Ussing chambers and treated with 1, 5, 10, 20, or 30 μM GM6001 for 30 minutes and stimulated with 45 BAU/mL trypsin. A representative tracing is shown in (**A**) and the percent change in TER 20 minutes post treatment with trypsin was determined from baseline taken as a percentage of the trypsin only control determined (**B**). No significant differences were observed comparing the groups to the untreated control, as determined by ANOVA. Cells were also pretreated with several concentrations of marimastat and TAPI-1 prior to apical trypsin treatment. Representative tracings are shown in (**C**) and (**E**) and summary data showing present change in TER are in (**D**) and (**F**). Like GM6001, no significant changes were observed in the response to trypsin with marimastat or TAPI-1.

Apical treatment with GM6001, an inhibitor of MMP-1, -2, -3, -8, -and -9, or marimastat, an inhibitor of MMP-1, -2, -3, -7, -8, and -14, for 30 minutes had no effect on the trypsin-mediated increase in TER at all doses investigated (Fig 4A–4D). A disintegrin and metalloproteinases (ADAMs) are closely related to MMPs and cleave membrane-bound ligands of the ErbB family to modulate signaling [28,29]. Since ADAM17 has been shown to promote intestinal epithelial restitution through transactivation of EGFR [30,31], it was hypothesized that this protein played a role in the serine protease-mediated increase in TER through EGFR. To test this hypothesis, SCBNs were pre-treated apically with 1, 5, 10, and 20 μM of the ADAM17 inhibitor TAPI-1 for 30 minutes, and then stimulated apically with 45 BAU/mL trypsin. No difference was observed between inhibitor-treated cells and control cells (Fig 4E and 4F).

### Trypsin-mediated initial increase in TER requires Src kinase activation

**Fig 5:**
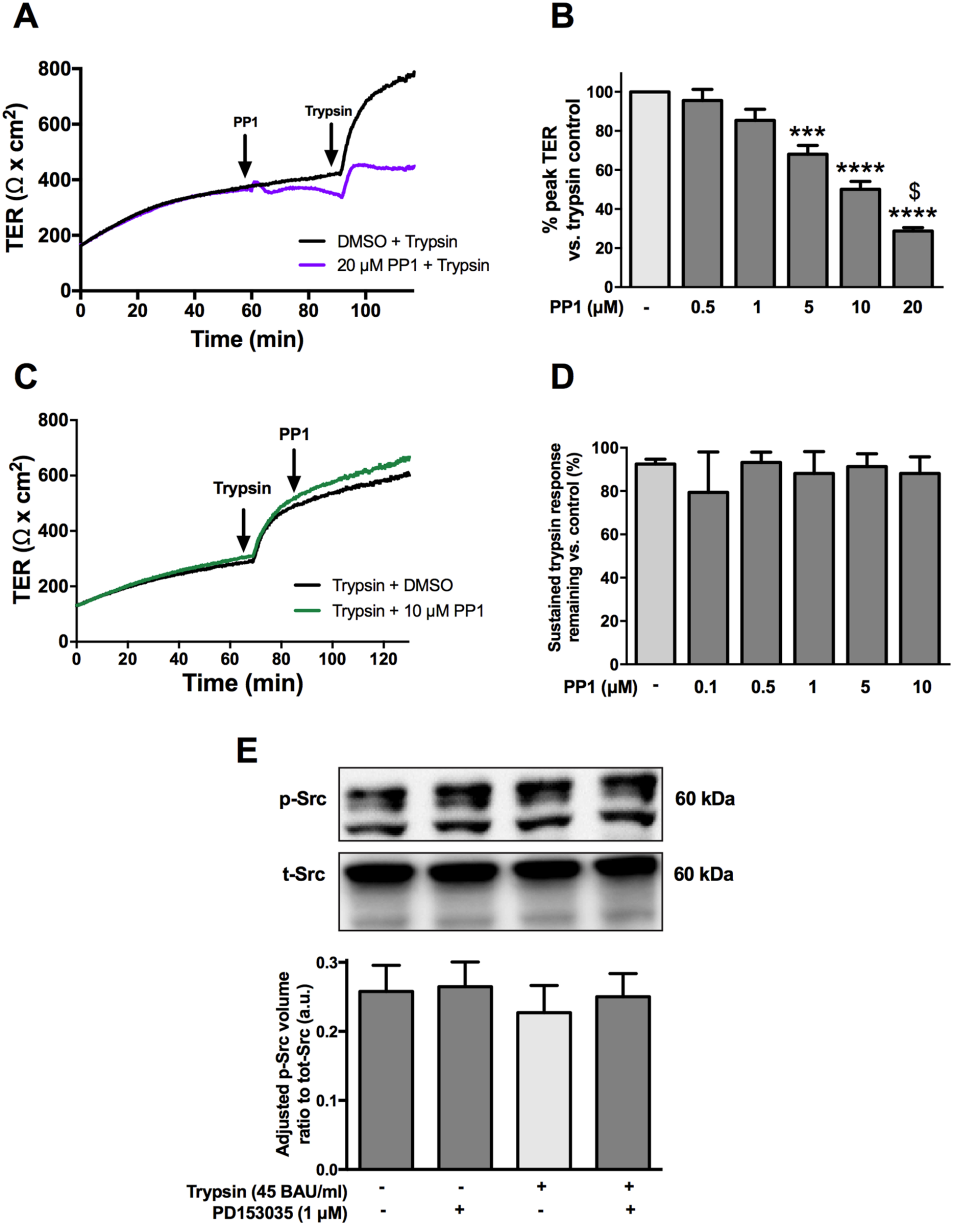
Src inhibition affects the initiation, but not sustained phase of the serine protease induced increase in TER. SCBN cells in Ussing chambers were treated with PP1 either 30 minutes before apical treatment with 45 BAU/mL trypsin or 10 minutes after trypsin challenge. Pretreatment with PP1 caused a reduction in the peak trypsin response as seen in the representative tracing in (**A**). The effect was dose dependent and quantified as the percent peak TER compared to the DMSO control in (**B**) (n = 3) *** p<0.001, ****<0.0001, $ p<0.05 vs 10 μM PP1 by ANOVA with Tukey’s post-hoc test. Treatment with PP1 after trypsin challenge resulted in no significant change in TER (n = 3). Representative tracing shown in **C**, and concentration response in **D**. SCBN cells grown on transwells were serum starved for 60 minutes with 1 μM PD153035 or DMSO and stimulated apically with 45 BAU/mL trypsin for 5 minutes. Cells were lysed and Src phosphorylation was determined via western blotting. Neither apical treatment with 45 BAU/mL trypsin, or treatment with PD153035 changed the phosphorylation of Src (**E**) (n = 3).

Src kinase has been shown to play a role in the regulation of barrier function [13,14]. Since the transactivation of EGFR can also occur through an intracellular Src kinase [32], we sought to determine if serine proteases induced an activation of EGFR to increase TER through Src. SCBN cells were pretreated with the Src inhibitor PP1 and then treated with 45 BAU/mL trypsin. PP1 dose-dependently blunted the peak response to trypsin (Fig 5A and 5B). However, the addition of PP1 10 minutes after apical trypsin stimulation had no effect on the sustained increase in TER (Fig 5C and 5D). Phosphorylation of Src was investigated by western blotting after both PD153035 (1 μM) and trypsin treatment. SCBN cells have a high baseline level of phosphorylated Src that was unchanged by serine protease or PD153035 treatment (Fig 5E).

### Trypsin-mediate increased TER is partially dependent on PI3-K signaling

**Fig 6:**
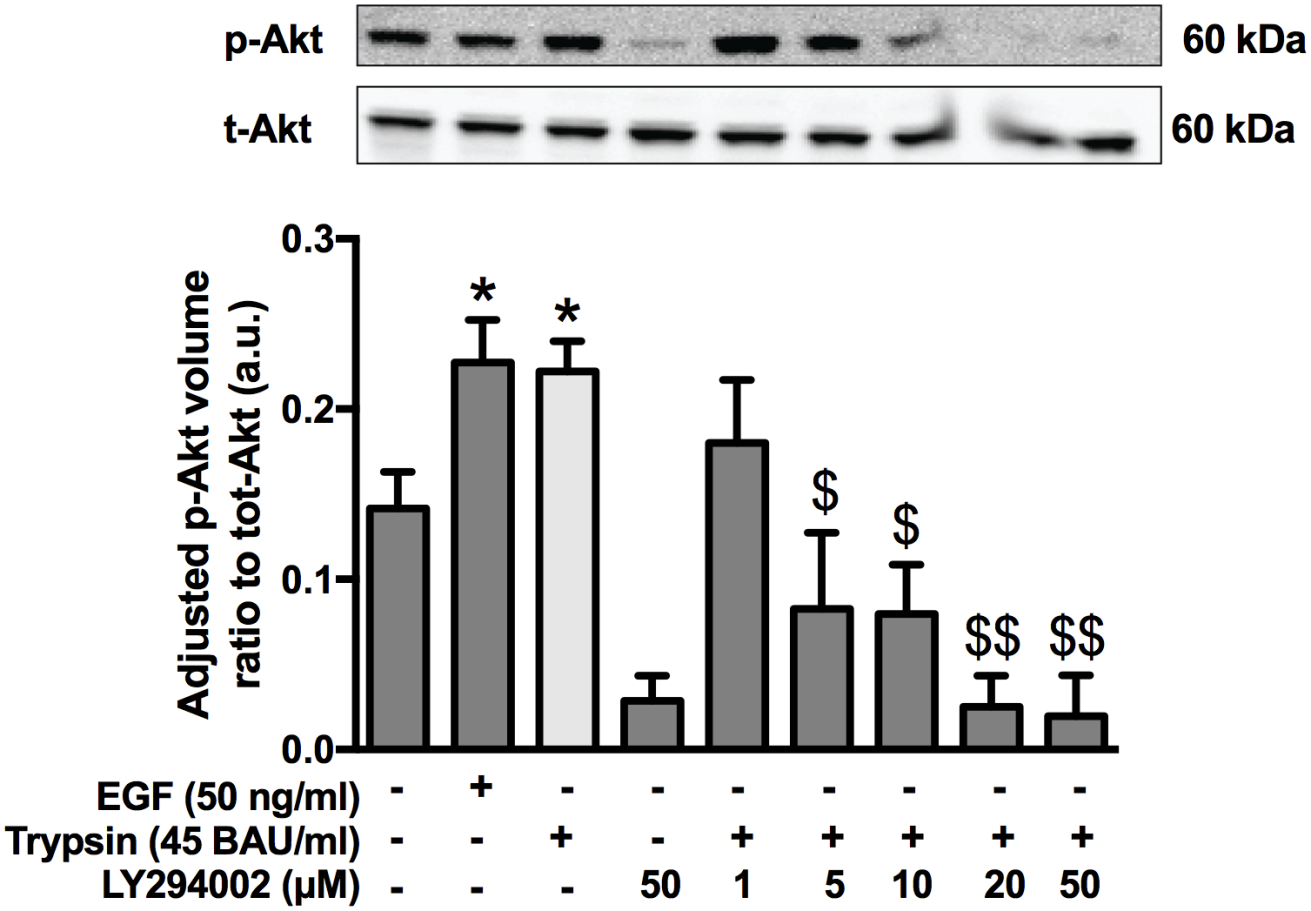
Trypsin induces phosphorylation of Akt. Confluent monolayers of SCBNs grown on transwells were serum starved for 60 minutes with increasing concentrations of LY294002 (1, 5, 10, 20, 50 μM) or DMSO then stimulated apically with 45 BAU/mL trypsin or 50 μg/mL EGF for 5 minutes. Cells were lysed and phosphorylated and total Akt levels assessed by Western blotting n = 3–5. Ratios of phosphorylated to total Akt densitometry values were calculated and normalized total fluorescence for each blot. *p<0.05 compared to non-treated control, $ p<0.001, $ $ p<0.0001 compared to trypsin alone. Analysis by ANOVA with Tukey’s post-hoc test.

EGFR is known to activate PI3-K, and therefore the involvement of EGFR in the protease-mediated increased TER was investigated. PI3-K activates Akt, a serine/threonine kinase known to regulate barrier function through modulation of tight junctional proteins, as well as the modulation EGFR signaling [33–35]. To determine the role of PI3-K in the serine protease mediated increase in TER, SCBN cells were pre-treated with LY294002 (1, 5, 10, 20, 50 μM), a selective inhibitor of PI3-K, or DMSO, then stimulated with 45 BAU/mL trypsin for 5 minutes. Cell lysates were collected and the phosphorylation of Akt determined by western blotting. Trypsin significantly increased the phosphorylation of Akt, comparable to that induced by EGF (50 ng/mL) (Fig 6). The phosphorylation induced by trypsin was prevented by LY294002 pretreatment.

**Fig 7:**
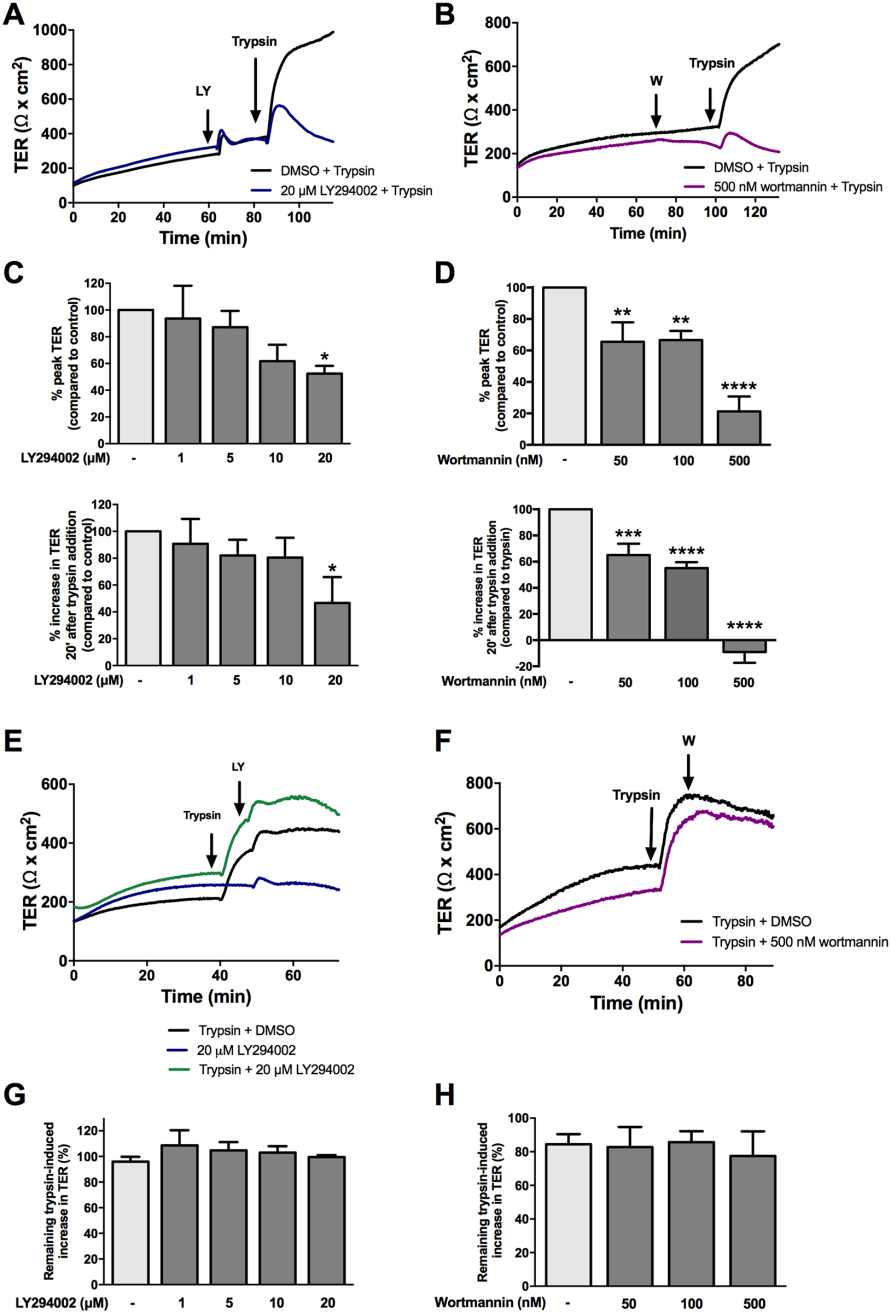
PI3K inhibition affects the initiation, but not sustained phase of the serine protease induced increase in TER. SCBN cells in Ussing chambers were pretreated with the PI3-K inhibitors LY293002 or wortmannin 30 minutes prior to apical challenge with 45 BAU/mL trypsin. Representative tracings are shown in (**A**) of n = 3–5 and (**B**) of n = 3–5, respectively. Data are presented as either the percent increase in TER 20 minutes after trypsin addition compared to control, or the percent peak TER, representing the peak and sustained TER. Data for LY294002 is shown in (**C**) and wortmannin in (**D**). Both LY294002 and wortmannin were also added during the sustained phases of the trypsin response, but no change in the response was observed. Representative tracings for LY294002 and wortmannin are shown in **E** and **F** and summary data in **G** and **H**, respectively, n = 3–4 for each.

In Ussing chambers, pretreatment with the PI3-K LY294002 at 20 μM decreased both the peak and sustained TER responses to trypsin (Fig 7A and 7C). However, adding LY294002 after the addition of trypsin did not significantly change the response to trypsin (Fig 7E and 7G). Since responses to trypsin following LY294002 exhibited variability between experiments, the irreversible PI3-K inhibitor wortmannin was also used. Pre-treatment with 50, 100, and 500 nM of wortmannin also decreased the peak and sustained response to trypsin (Fig 7B and 7D). Cells were also treated with wortmannin during the sustained phase, and it was found that, like LY294002, it had no effect on the TER change (Fig 7F and 7H).

### Protease-mediated increase in TER is partially dependent on ERK1/2 but not p38 MAPK signaling

**Fig 8:**
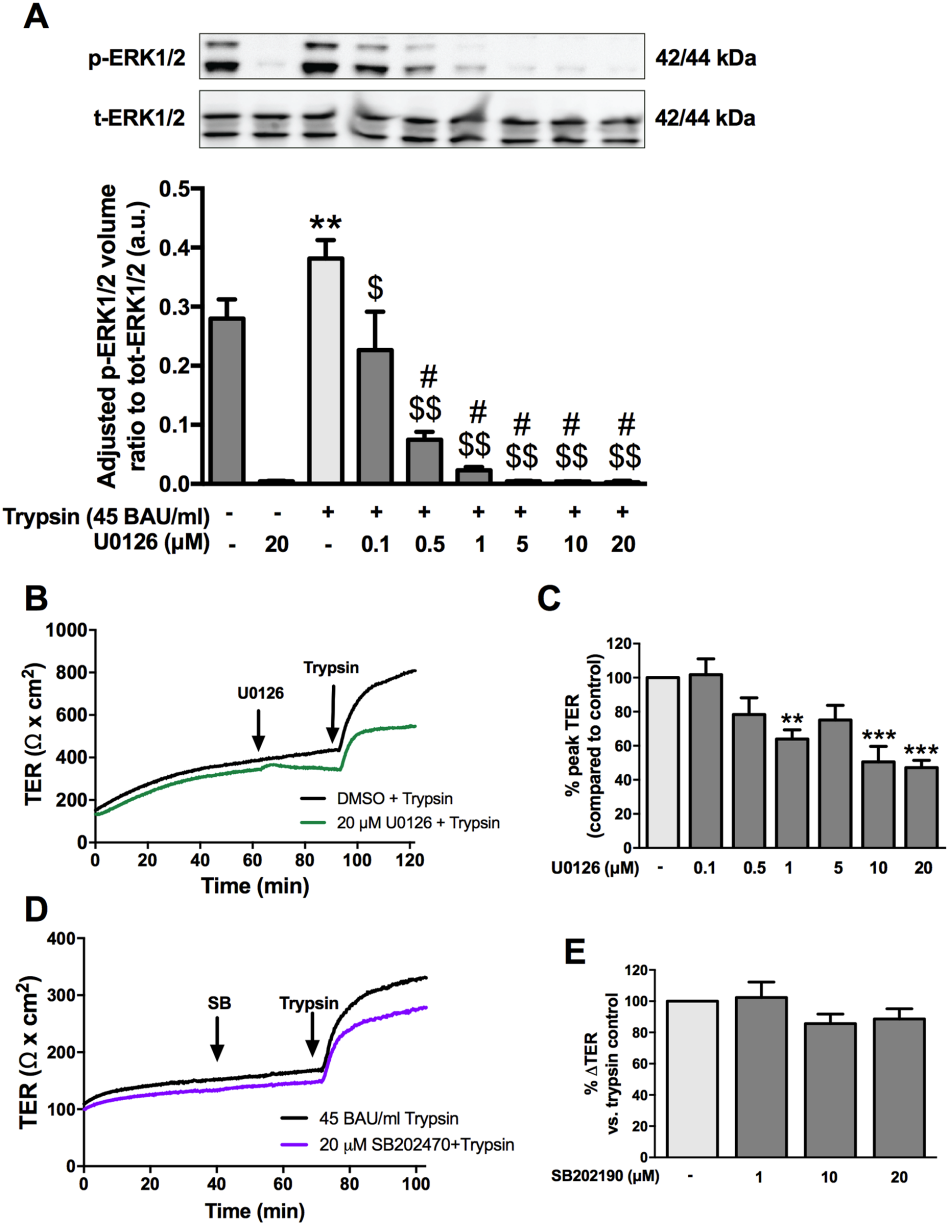
The roles of ERK1/2 and p38 in the serine protease mediated increase in TER. Confluent monolayers of SCBNs grown on transwells were serum starved for 60 minutes with increasing concentrations of U0126 (0.1, 0.5, 1, 5, 10, 20 μM) or DMSO and then stimulated apically with 45 BAU/mL trypsin or 50 μg/mL EGF for 5 minutes. Cells were lysed and phosphorylated and total ERK1/2 levels assessed by Western blotting. **A**. Representative blots of phosphorylated and total ERK1/2, n = 3. Ratios of phosphorylated to total ERK1/2 densitometry values were calculated and normalized to total fluorescence for each blot. ** p<0.01 compared to non-treated DMSO control, $ p<0.001, $ $ p<0.001 compared to trypsin, # p<0.0001 compared to 0.1 μM U0126 + trypsin. Analysis by ANOVA with Tukey’s post-hoc test. Cells in Ussing chambers were pretreated with various concentrations of U0126 and SB202470 and representative tracings shown in (**B**) (n = 3–4) and (**D**) (n = 3). **C**. Percent peak TER compared to control was assessed for treatment with U0126 and significant differences were found for 1, 10, and 20 μM. ** p<0.01, *** p<0.001 compared to DMSO + trypsin controls by ANOVA with Dunnett’s post-hoc test. **E**. No significant differences were observed for percent change in TER in cells pretreated with SB20240 and challenged with trypsin.

ERK1/2 is downstream of EGFR and modulates barrier function through phosphorylation of tight junctional proteins [11]. Since the response to serine protease was dependent on EGFR, downstream signaling through ERK1/2 was then examined. SCBN cells were treated with the MEK1/2 inhibitor, U0126 (0.1, 0.5, 1, 5, 10, and 20 μM), and then treated with trypsin for 5 minutes. Cells were collected and analyzed for the phosphorylation of ERK1/2 via western blotting. Trypsin treatment significantly increased phosphorylation of ERK1/2 (Fig 8A). U0126 decreased the constitutive phosphorylation of ERK1/2 in SCBN cells, and inhibited the trypsin-induced phosphorylation.

In Ussing chambers, SCBN cells were treated with 0.1, 0.5, 1, 5, 10, or 20 μM U0126 for 3 minutes then stimulated with 45 BAU/mL trypsin. 1, 10, and 20 μM U0126 significantly decreased the peak TER (Fig 8B and 8C). Another arm of the MAPK signaling pathway, p38 MAPK, was also investigated for its role in the serine protease-induced increase in TER. Apical pre-treatment with the p38 inhibitor SB202470 at 1, 10, or 20 μM had no effect on the sustained increase in TER following apical trypsin treatment (Fig 8D and 8E).

### The serine protease-mediated increase in TER is dependent on CK2

**Fig 9:**
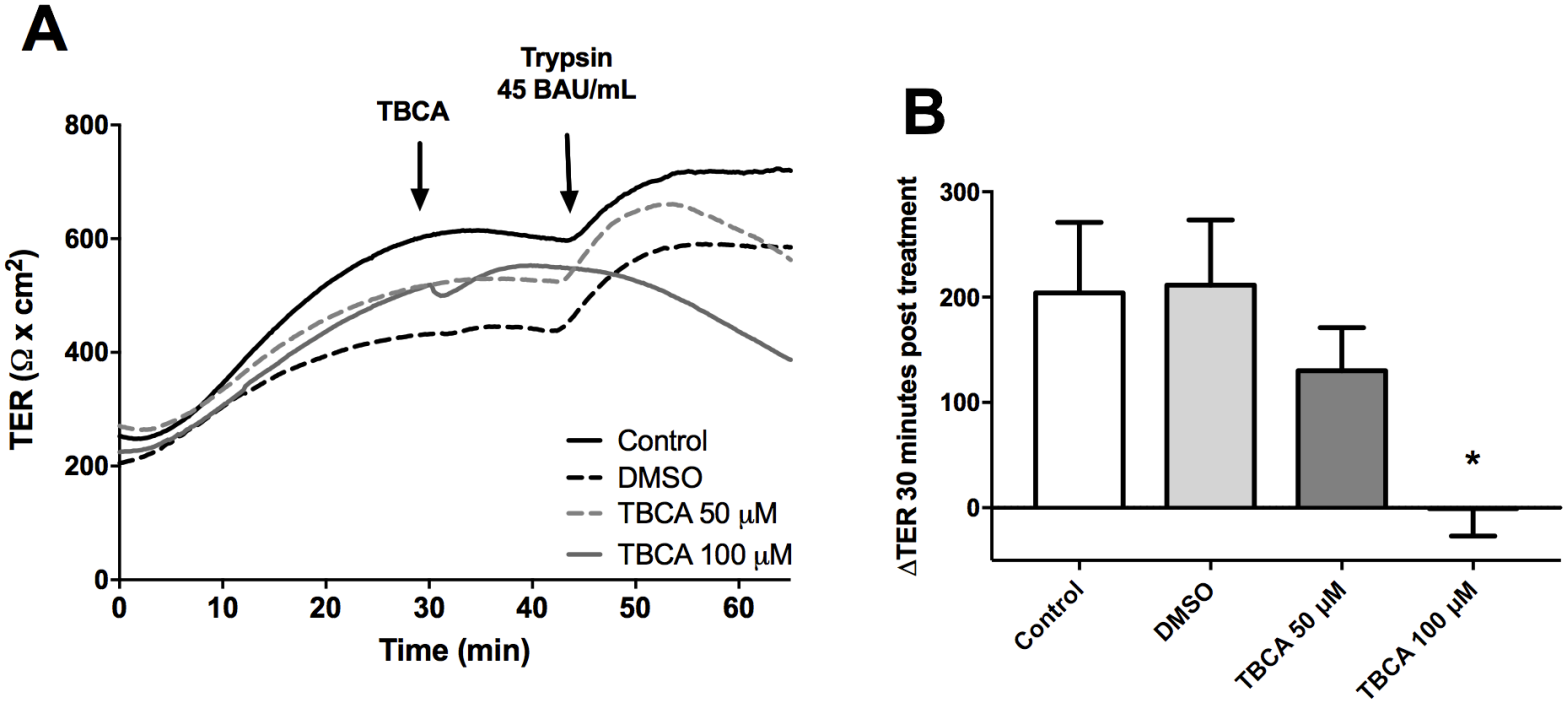
Inhibition of CK2 completely prevents the serine protease induced increase in TER. SCBN cells were mounted in Ussing chambers and treated apically with 50 or 100 μM TBCA for 30 minutes prior to apical challenge with 45 BAU/mL trypsin. A representative tracing is shown in **A**. The peak change in TER post trypsin was determined and TBCA at 100 μM significantly reduces the change in TER in response to trypsin (**B**). * p<0.05 as assessed by ANOVA with Dunnett’s post hoc test compared to DMSO control. N = 5–8.

Previous work has determined that CK2 can modulate barrier function in epithelial cells through effects on phosphorylation of tight junction proteins [18,36]. SCBN cells were pretreated with the CK2 inhibitor TBCA at 50 or 100 μM for 10 minutes before being treated apically with 45 BAU/mL trypsin. CK2 inhibition with TBCA at 100 μM prevented the increase in TER induced by trypsin (Fig 9A and 9B).

### The protease-mediated increase in TER does not involve activation of bradykinin B(2) receptor

**Fig 10:**
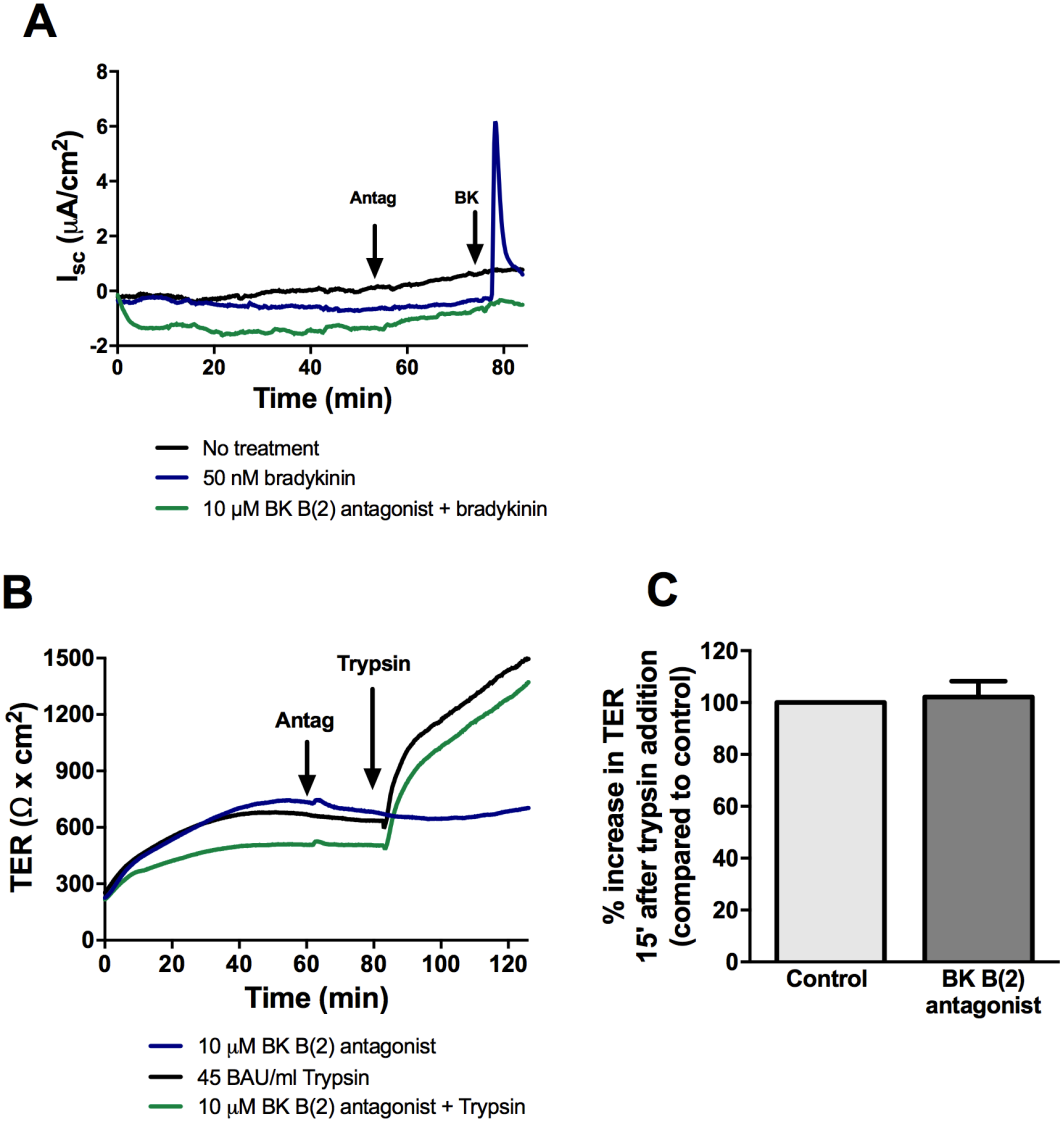
Inhibition of bradykinin B(2) receptor does not affect the trypsin mediated increase in TER. **A**. SCBN cells were mounted in Ussing chambers and treated basolaterally with the bradykinin B(2) receptor antagonist (10 μM) for 30 minutes then treated basolaterally with 50 nM bradykinin to determine the lowest dose of antagonist that blocks the chloride secretion. **B**. SCBN cells in Ussing chambers were treated apically with 10 μM antagonist for 30 minutes then stimulated apically with 45 BAU/mL trypsin. Tracing representative of n = 3. **C**. Percent increase in TER 15 minutes after trypsin addition normalized to the uninhibited control were determined. One sample T-test indicated no significant differences.

Since EGFR inhibition was shown to only partially reduce the change in TER induced by serine protease, it suggests other cell surface targets are involved. The bradykinin B(2) receptor can be directly activated by tissue and plasma kallikreins, cathepsins, and trypsin [37]. Bradykinin is known to induce chloride secretion, and basolateral treatment of SCBN cells in Ussing chambers with bradykinin resulted in an increase in short circuit current, indicative of chloride secretion and presence of bradykinin receptors. SCBNs were pre-treated with increasing doses of bradykinin B(2) receptor agonist to determine the lowest effective concentration that inhibited chloride secretion (10 μM, Fig 10A). Apical pre-treatment with B(2) antagonist for 30 minutes had no effect on trypsin-induced barrier enhancement (Fig 10B and 10C).

### The serine protease mediated increase in TER is not dependent on MLC phosphorylation

It was previously determined that the serine proteases trypsin and matriptase cannot reverse disruption of the tight junction induced by the inflammatory cytokines IFNγ and TNFα [22]. The inability of serine proteases to change TER after cytokine treatment may be due to an increase in MLC phosphorylation. It was hypothesized that a decrease in permeability would be accompanied by a decrease in MLC phosphorylation.

**Fig 11:**
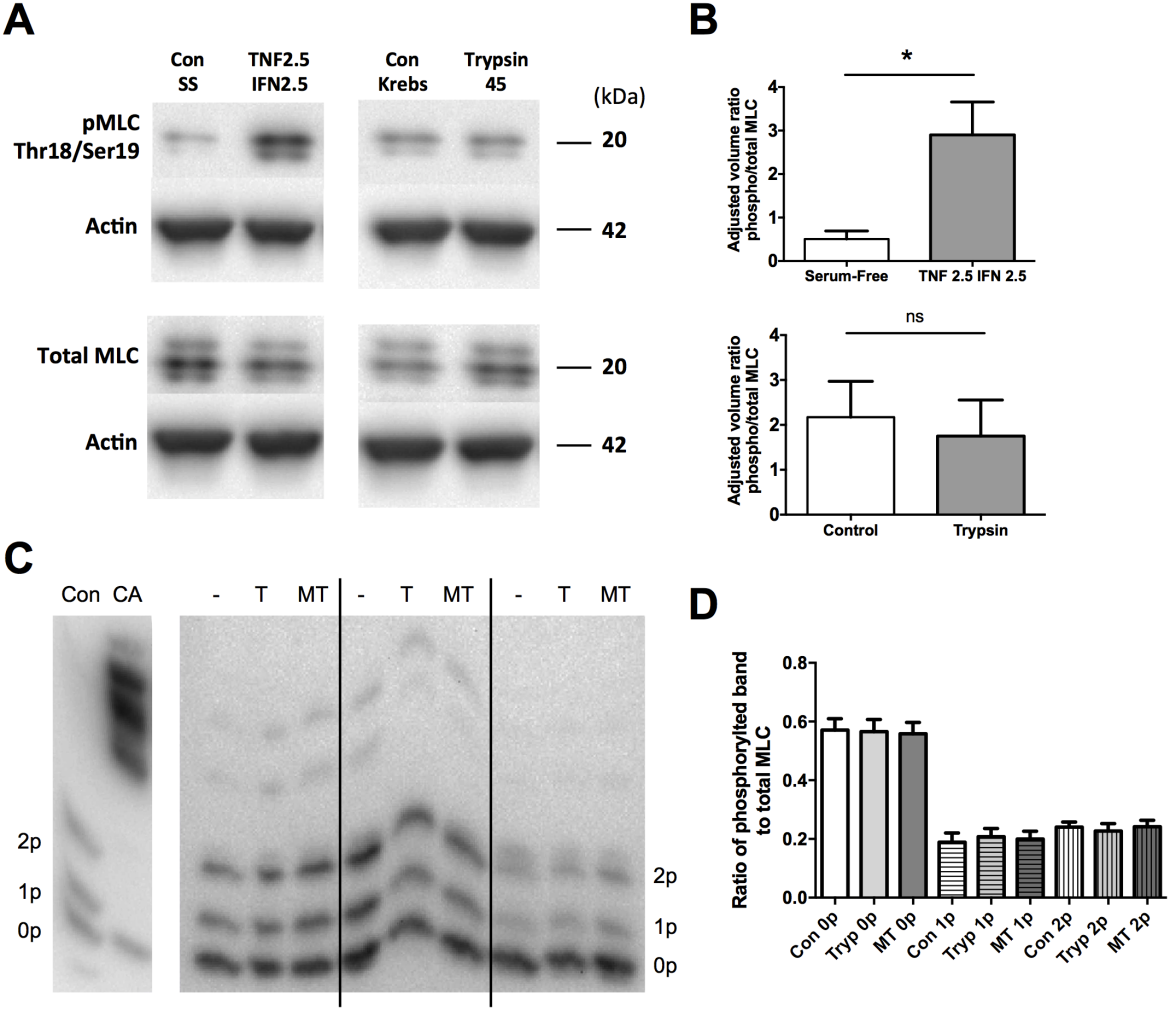
Serine proteases do not induce an increase in phosphorylation of myosin regulatory light chain. Phosphorylation of MLC was assessed using phospho-specific antibodies (Ser19/Thr18) and western blotting **A**. A representative blot of phosphorylated and total MLC. **B**. Densitometry was performed with normalization to actin and the ratios of phosphorylated to total MLC were determined. n = 3–4; ns, not significant compared to control as analyzed by ANOVA with Dunnett’s posthoc test. **C**. MLC phosphorylation was also determined by Phos-tag gels. SCBN cells were plated on transwells and treated for 15 minutes apically in Krebs with 135 BAU/mL trypsin (T) or 1.5 BAU/mL matriptase (MT). Lysate was collected and phosphorylation of MLC assessed by Phos-tag gel electrophoresis. Protein with zero, one, or two phosphorylations (0p, 1p, 2p) are labeled. The positive control is SCBN cells treated with 3 μM of the phosphatase inhibitor calyculin A (CA) apically for 30 minutes. A blot with n = 3 is shown in **C**, while densitometry is shown in **D** and is the ratio of the phosphorylated band over the total MLC (all three bands combined), n = 6. No significant differences are seen as assed by ANOVA within each phosphorylated group.

To determine MLC phosphorylation, two methods were employed. The first was the measurement of phosphorylated MLC using specific antibodies to the Thr18/Ser19 phosphorylation site of MLC. SCBN cells were treated for 48 hours with 2.5 ng/mL IFNγ and 2.5 ng/mL TNFα, as this dose was previously found to induce barrier disruption without apoptosis [22,38]. This experiment showed that treatment with cytokines caused an increase in phosphorylated MLC in SCBN cells (Fig 11A). Having confirmed that MLC phosphorylation could be increased this cell line, subsequent experiments were performed where SCBN cells were treated apically for 15 minutes with 45 BAU/mL trypsin. No changes in MLC phosphorylation were observed with trypsin treatment (Fig 11B).

To verify these results, MLC phosphorylation was also investigated using Phos-tag western blotting. Using this technique, MLC protein resolves into 3 bands, representing MLC that is phosphorylated at 0, 1, or 2 residues. Some higher phosphorylated bands were also observed. SCBN cells were treated for 15 minutes apically with trypsin (45 BAU/mL) or matriptase (0.5 BAU/mL) and phosphorylation pattern assessed (Fig 11C). No changes in any of the bands were observed (Fig 11D). Thus, neither trypsin nor matriptase induce the phosphorylation or dephosphorylation of MLC.

## Discussion

Recent literature suggests an important role for the endogenous epithelial serine proteases matriptase and prostasin in establishing and maintaining the intestinal epithelial barrier [23,24,39,40]. The apical addition of serine proteases has been shown to significantly increase TER in several colonic epithelial cell lines in an activity-dependent manner [21]. In that study, we showed an important but partial role for PCKζ. Other intracellular mechanisms underlying this response remained unknown. Thus, the main objective of the present study was to determine the downstream signaling events governing protease-mediated barrier enhancement in intestinal epithelial cells.

We found that the trypsin- and matriptase-induced increases in barrier function were dependent on catalytic activity of the protease. Pretreating cells with SBTI prevented the increase in TER induced by trypsin. Interestingly, inhibition of trypsin or matriptase during the sustained phase of the response induced an immediate and rapid decrease of TER. This suggests that ongoing cleavage of a receptor or ligand from the surface is needed to induce and maintain the effect. There is no significant delay in the decrease, which suggests any signal transduction pathways induced by serine proteases are rapidly switched on or off.

The apical addition of EGF significantly increased TER in SCBN monolayers compared to non-treated controls. The fact that EGF exhibited similar effects on TER as trypsin suggested that serine protease-mediated barrier enhancement could be occurring through EGFR signaling. EGF-mediated epithelial barrier enhancement has been documented in other cell lines, namely MDCK [41,42], and the prostasin-matriptase cascade is able to cleave the extracellular domain of EGFR to form a truncated receptor with tyrosine kinase activity and altered signaling [43]. These observations suggest that serine proteases may cleave a growth factor such as EGF from the cell surface to induce an increase in TER.

We investigated the effect of EGFR tyrosine kinase inhibition on the protease-mediated increase in TER using PD153035. Inhibiting EGFR prior to trypsin stimulation altered the overall response; although TER increased with the same slope and in the same time frame after trypsin treatment, the maximal increase was blunted and the sustained phase was significantly decreased in inhibitor-treated cells, suggesting some requirement for EGFR activation in the persistence of the protease-mediated TER response. The addition of the EGFR inhibitor PD153035 10 minutes after trypsin stimulation, when the initiation phase was tapering to a plateau, significantly decreased the sustained phase of the response. Unlike the immediate decrease in TER seen following SBTI treatment, PD153035 treatment did not elicit a decrease until approximately 20 minutes after its addition. This is likely due to the requirement for PD153035 to get through the cell membrane and into the intracellular receptor domain prior to having an effect [44]. Initiation of the trypsin response still occurred in the presence of PD153035, albeit at a smaller magnitude than in control trypsin-treated cells. This suggests that EGFR is not being activated directly by proteases, but is likely engaged through a transactivation mechanism, or other cell surface receptors are involved in the response.

Although matriptase has been shown to directly cleave the extracellular domain of EGFR [43], the EGFR inhibitor experiments point to a transactivation mechanism for EGFR following protease stimulation. Two well-known mechanisms of EGFR transactivation are: (1) extracellular cleavage of membrane-bound EGFR ligands by activated MMPs or ADAMs that can then engage the receptor, and (2) intracellular phosphorylation of EGFR tyrosine residues by Src kinases [27,32]. We determined the involvement of MMP-mediated cleavage and of ADAM-17 in the serine protease induced increase in TER. Pre-treatment with broad spectrum MMP inhibitors or the ADAM-17 inhibitor TAPI-1 had no effect on trypsin-induced increased TER at all doses investigated, suggesting MMP-dependent extracellular release of EGFR ligands and subsequent EGFR activation does not occur in trypsin-induced increased TER.

Intracellular, Src-mediated EGFR transactivation was hypothesized to be one potential mechanism by which trypsin increases TER in SCBNs. We showed that pre-treatment with the Src inhibitor, PP1, decreased trypsin-induced increased TER in a concentration-dependent fashion. The addition of PP1 10 minutes after trypsin stimulation did not decrease trypsin-induced sustained TER. Thus Src is involved in the initiation, but not sustained phase of the serine protease induced increase in TER. Src phosphorylation was not changed by serine protease treatment, but the levels of endogenous phosphorylation were high, as we have observed for several signaling proteins in unstimulated SCBN cells (unpublished observations). It may be that subtle changes in phosphorylation status following addition of pharmacological inhibitors are sufficient to elicit and change in TER, but which may not be detected by western blotting.

EGFR, ErbB3, and ErbB4 contain PI3-K-specific tyrosine residues, and ErbB signaling is known to activate PI3-K [34]. PI3-K activation has been shown to modulate tight junctional proteins, claudin-1 and -2, to increase permeability in HT-29 and Caco-2 cell monolayers [33,35]. Trypsin stimulation significantly increased Akt phosphorylation, suggesting PI3-K activation is increased by this protease. We used the PI3-K inhibitors, LY294002 and wortmannin, to investigate the effects of PI3-K on protease-mediated increased TER. PI3-K inhibition prior to trypsin stimulation significantly decreased the peak increase and sustained TER compared to trypsin-treated cells, suggesting PI3-K involvement in both phases of the trypsin response. However, inhibiting PI3-K in the sustained phase of the trypsin response (after trypsin stimulation) had no effect on the sustained response. These data, together with the EGFR inhibition data suggest PI3-K is likely involved only in the initiation phase of the response. Because EGFR is required for the sustained trypsin response, and PI3-K is likely not, PI3-K (and not EGFR) signaling may be activated by the initial cell surface target cascade.

ERK1/2 signaling has been shown to modulate barrier function. The previously observed ouabain-induced increase in TER occurs through EGFR and ERK1/2 signaling in MDCK cells [45]. IFNγ mediates increased bacterial translocation across T84 colonic epithelial cells through an ERK1/2-dependent mechanism, therefore ERK1/2 phosphorylation can have both positive and detrimental effects on intestinal epithelial barrier function [46]. Trypsin stimulation induced a significant increase in phosphorylated ERK1/2 that was blocked by the MEK1/2 inhibitor, U0126. Pre-treatment with U0126 decreased the maximal increase in TER following trypsin stimulation, suggesting a requirement for ERK1/2 in initiating protease-mediated enhanced barrier function. Like PI3-K, this finding suggests an EGFR-independent activation of ERK1/2 in the protease-mediated response, as EGFR was implicated in the sustained phase of the trypsin response. p38 is another MAPK signaling pathway that can be activated by growth factors. However, p38 MAPK inhibition by SB202470 had no effect on protease-mediated increased TER, suggesting a p38-independent mechanism by which proteases enhance barrier function in the intestinal epithelium.

CK2 is a kinase previously shown to regulate binding of occludin with ZO-1 and claudin family members through phosphorylation of occludin and to affect barrier function [18,36]. We inhibited CK2 using the inhibitor TBCA. Because TBCA is insoluble in buffer containing magnesium, Ussing chamber experiments were performed in Krebs buffer lacking MgCl_2_. Pretreatment with this CK2 inhibitor significantly reduced the trypsin-induced increase in TER. In the study by Raleigh *et al*, CK2 inhibition by TBCA in Caco-2 cells induced an increase in TER. However, we observed a decrease in TER in SCBN cells treated with TBCA. One reason for this is that SCBN cells do not express claudin-2 [22], which is affected by CK2-induced phosphorylation of occludin at S408. Thus there may be differential mechanisms on the junction by CK2 when claudin-2 is not expressed. More work is needed to determine how CK2 phosphorylates occludin or other tight junction proteins in SCBN cells.

Because cytokines have been shown to induce both the internalization of tight junctional proteins and a phosphorylation of myosin light chain to increase permeability, we examined whether trypsin and matriptase impact the dephosphorylation of MLC. Dephosphorylation through myosin light chain phosphatase has been demonstrated in smooth muscle tissues to induce relaxation [47]. While epithelial cells express MLCP [48], its role in regulating barrier function has not been determined. MLC phosphorylation was determined through two means, either through western blotting of phosphorylated MLC at Thr18/Ser19, or through Phos-tag gels. Increased phosphorylation at Thr18/Ser19 was observed when SCBN cells were treated with cytokines, as expected. However, no change in phosphorylation was observed with either method when cells were treated apically with trypsin or matriptase. Thus these proteases do not produce an effect on barrier function through modulation of myosin light chain.

EGFR inhibition did not fully block the response to trypsin, suggesting some other cell surface receptor is the initial protease target that mediates initiation of increased TER. One potential target was the bradykinin B(2) receptor, which has been shown to modulate tight junction permeability through an ADAM17-EGFR transactivation mechanism that results in ZO-1 rearrangement [49]. Kallikreins, trypsin, and plasmin are known to indirectly activate bradykinin B(2) receptor through cleavage of high molecular weight kininogen that releases the 9-amino acid peptide bradykinin (or other kinins) to engage the receptor [50]. More recently, proteases were shown to directly activate bradykinin B(2) receptor independently of ligand-cleavage mechanisms [37]. The bradykinin B(2) receptor has been linked to increased severity of disease in murine models of DSS-induced colitis through modulation of the tight junction, suggesting a B(2) receptor-mediated effect on epithelial permeability [51]. Pre-treatment with the bradykinin B(2) receptor antagonist at the concentration that effectively blocked bradykinin-mediated chloride secretion had no effect on trypsin-mediated increased TER, suggesting that the B(2) receptor is not the initial cell surface target of trypsin.

**Fig 12:**
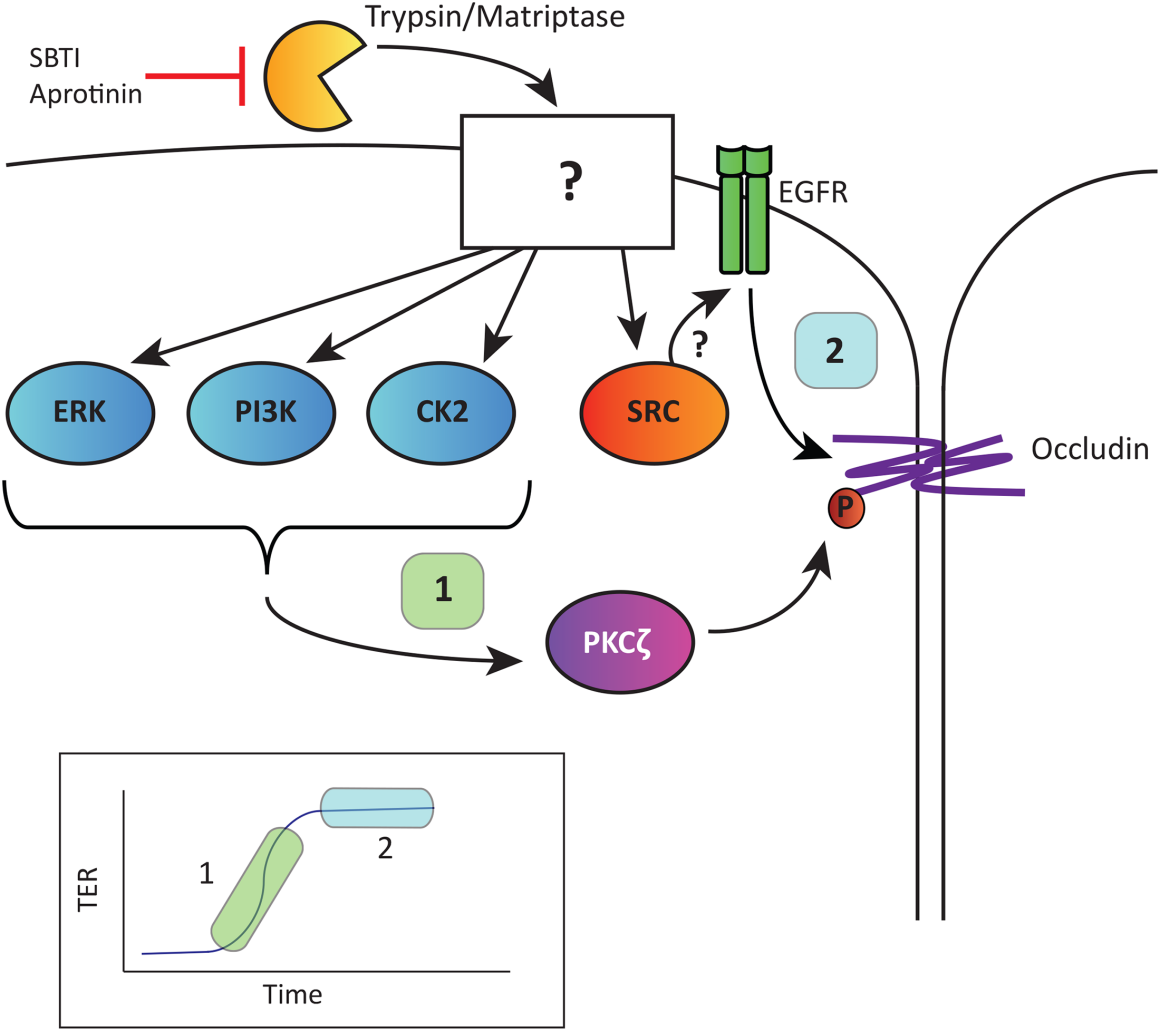
Summary of signaling pathways mediating the increased TER induced by apical trypsin in SCBN cells. Serine proteases (trypsin, matriptase) cleave an as yet unidentified surface molecule (or molecules) that trigger a rapid and sustained increase in TER (inset). The initiation phase **(1)** involves ERK1/2, PI3-K, CK2 and Src. The sustained phase **(2)** is partly dependent on EGFR tyrosine kinase activity which may be due to a Src-dependent EGFR transactivation. Our previous studies [20] demonstrated a downstream role for PKCζ-dependent phosphorylation of occludin. Indeed, occludin is the key tight junction protein responsible for the protease-mediated increase in TER [22].

Taken together, our results show that the serine protease-induced increase in TER can be divided into two phases, the initiation of the change in TER and the sustained phase where the TER remains elevated. The initiation phase is dependent on ERK1/2, Src, PI3-K, CK2, and the sustained phase is dependent on EGFR (Fig 12). We also show that the trypsin induced increase in TER is not dependent on cleavage of MMPs, MLC phosphorylation or bradykinin B(2) receptor activation. More work will be needed to determine how these signaling pathways affect phosphorylation of tight junctional proteins such as occludin to modulate barrier function, however. By elucidating mechanisms by which epithelial barrier function is regulated, our work could contribute to the discovery of novel therapeutic targets for intestinal inflammatory diseases, such as IBD, that are characterized by a disrupted epithelial barrier.
